# Definition of Polypharmacy in Atrial Fibrillation and Atrial Flutter: A Scoping Review

**DOI:** 10.14740/cr2236

**Published:** 2026-07-17

**Authors:** Ali Murra, Akhil Mahant, Lorraine Porcello, Burr Hall, Keshav Patel

**Affiliations:** a Department of Internal Medicine, University of Rochester Medical Center, Rochester, NY, USA; b University of Rochester School of Medicine and Dentistry, Rochester, NY, USA; c Edward G. Miner Library, University of Rochester Medical Center, Rochester, NY, USA; d Department of Cardiology, University of Rochester Medical Center, Rochester, NY, USA

**Keywords:** Polypharmacy, Atrial fibrillation, Atrial flutter, Drug-drug interactions, Medication burden

## Abstract

Polypharmacy lacks a consistent definition in the literature and studies use varying numeric thresholds, ranging from the use of four to as many as 15 medications with the most frequent cutoff being five or more medications. It is highly prevalent and particularly relevant in patients with atrial fibrillation (AF) and atrial flutter (AFL) who have multiple comorbidities and often require multi-agent therapy. As a result, these patients face increased risk of bleeding, falls, drug-drug interactions, adverse drug-related events, and medication non-adherence. This scoping review aims to identify how polypharmacy is defined in AF and AFL, to examine the clinical consequences of these definitions, and summarize considerations to prevent polypharmacy. A comprehensive literature search was completed across five databases to identify articles that address and define polypharmacy in AF and AFL. A protocol was prospectively registered on PROSPERO (CRD420251135482) before data extraction began. Included articles defined polypharmacy in adult patients with a diagnosis of AF or AFL. Of the 109 articles identified, 96% (n = 105) discussed AF only, 4% (n = 4) discussed AF and AFL together without differentiation, and none focused solely on AFL. Most articles (96%, n = 105) used a quantitative definition, while 4% (n = 4) used a qualitative definition. The most common definition of polypharmacy, reported in 51% (n = 56) of articles, was the use of five or more medications. This definition is not clinically meaningful as most patients with AF or AFL are already on five or more medications. There is ongoing debate on the definition of polypharmacy in this population. If a numeric cutoff is utilized, we propose increasing it to 10 or more medications as has been suggested in other cardiovascular conditions such as heart failure. More importantly, regardless of how we define polypharmacy in AF or AFL, it is crucial that providers continually assess each medication to ensure that the benefits outweigh the risks.

## Introduction

Atrial fibrillation (AF) and atrial flutter (AFL) are arrhythmias affecting approximately 5% of US adults [1, 2]. The prevalence and lifetime risk of AF and AFL increases with age, posing a growing public health challenge [2]. Management of AF and AFL includes lifestyle modification and a multifaceted pharmacotherapy approach to decrease arrhythmia burden, improve symptoms, and improve quality of life [3, 4]. Clinicians prescribe beta-blockers or calcium channel blockers to control the heart rate and class I and class III antiarrhythmics are used to maintain sinus rhythm [3]. Anticoagulants are used for ischemic stroke prevention in patients with elevated risk [3, 4]. Greater than 80% of patients with AF have two additional comorbid conditions, with hypertension (HTN), coronary artery disease (CAD), and heart failure (HF) being the most common, all of which require additional medical management [5, 6]. Therefore, patients with AF/AFL are at a high risk of polypharmacy.

Polypharmacy can lead to drug-drug interactions, adverse drug events, falls, dementia, frailty, and reduced drug compliance [7]. There have been numerous initiatives to help reduce polypharmacy and inappropriate prescribing, particularly in elderly patients. The Beers Criteria is a list of medications that should be avoided in adults > 65 years of age to limit potentially inappropriate medications with higher risk of adverse events or side effects in the aging population [8]. Several medications on this list are prescribed to patients with AF and AFL, including rate control agents such as digoxin, rhythm control agents such as dronedarone and amiodarone, and anticoagulants such as rivaroxaban and warfarin [8]. Another tool that guides prescribing practices in older adults is the Screening Tool of Older Person’s Prescriptions (STOPP) and Screening Tool to Alert to Right Treatment (START) criteria [9]. The STOPP criteria addresses medications that could be harmful or result in adverse effects in older adults. The START criteria involves recommendations for medications that should be prescribed to modify or prevent disease in the older population. Using STOPP/START criteria reduces polypharmacy, incorrect dosing, adverse drug reactions, and drug-drug interactions [9]. Although these indices focus on high-risk medications, medications that are considered relatively safe and otherwise have a low risk of adverse effects, such as melatonin or vitamin D, also contribute to polypharmacy.

Beyond these tools, arrhythmia-specific non-pharmacologic treatments for AF or AFL may also reduce polypharmacy. All patients should undergo diet and lifestyle counseling, and providers should emphasize the importance of exercise, alcohol avoidance, and weight loss in overweight individuals. A subset of patients may also benefit from procedures such as cardioversion and catheter ablation to terminate the arrhythmia and left atrial appendage occlusion (LAAO) to minimize stroke risk and de-escalate anticoagulation (AC).

Definitions of polypharmacy vary in the literature and include both quantitative thresholds and qualitative assessments. Polypharmacy is a critical issue in this patient population as these patients are older, more likely to be on AC, and have an increased fall risk. This scoping review aims to identify the existing definitions of polypharmacy in AF and AFL patients, examine the clinical implications, and identify areas where deprescribing may be clinically indicated.

## Methods

### Protocol and reporting

We prospectively registered a protocol in PROSPERO (CRD420251135482) and reported the review in accordance with PRISMA-ScR guidance [10].

### Eligibility criteria and conceptual framework

This is a scoping review, therefore the population–concept–context (PCC) framework was used to conceptualize the research question and structure the search in modular components. The population included adults with AF and/or AFL; the concept was polypharmacy; and the context encompassed any clinical or research setting [10].

### Information sources

A comprehensive literature search was conducted in MEDLINE (Ovid), Embase (Elsevier), CINAHL (EBSCOhost), The Cochrane Library (Cochrane Database of Systematic Reviews, Cochrane Protocols, and CENTRAL), and Web of Science (All Databases). Within Web of Science, the search queried multiple segments via the All Databases option, including the Web of Science Core Collection and specialty indexes (e.g., BIOSIS Citation Index, Current Contents Connect, KCI – Korean Journal Database, MEDLINE, Preprint Citation Index, and SciELO Citation Index), as enabled by local institutional access [11].

### Search strategy development and translation

The initial strategy was developed in MEDLINE (Ovid) by a health sciences librarian (LP) in collaboration with the subject-matter experts (KP, AM) using 17 seed articles supplied by the team to ensure sensitivity to known relevant literature. The search was constructed modularly to reflect the PCC elements and combined with the Boolean operator AND. After team consensus on the MEDLINE strategy, Polyglot (Systematic Review Accelerator) was used to efficiently translate the strategy to other databases; each translation was then reviewed and, where needed, edited by the librarian to account for database-specific syntax and subject headings [12].

No language, study-design, or publication-type limits were applied in any database. All databases were searched from inception through August 26, 2025. Complete, reproducible search strategies for every database are provided in Supplementary Material 1 (cr.elmerpub.com).

### Study selection workflow and data management

All search results were imported into Covidence (Veritas Health Innovation, Melbourne, Australia) for de-duplication and screening workflow management (title/abstract and full-text). Automatic deduplication was performed using Covidence’s default algorithm, followed by manual review of remaining potential duplicates. Within The Cochrane Library, only records retrieved from CENTRAL were imported into Covidence for screening (records from CDSR and Cochrane Protocols were reviewed as contextual sources but not screened as primary studies) [13].

### Screening and data extraction

Eligible materials included published original research, reviews, guidelines, case reports, abstracts, editorials, and consensus statements. We included articles that focused on adult patients and included a definition of polypharmacy in AF and AFL. AF and AFL were considered together because these arrhythmias share overlapping etiologies, risk factors, and pharmacologic therapies, although important differences in long-term management exist. Articles were excluded if they were written in a non-English language, patients did not have AF or AFL, there was no definition of polypharmacy, or if subjects < 18 years old were included.

**Figure 1:**
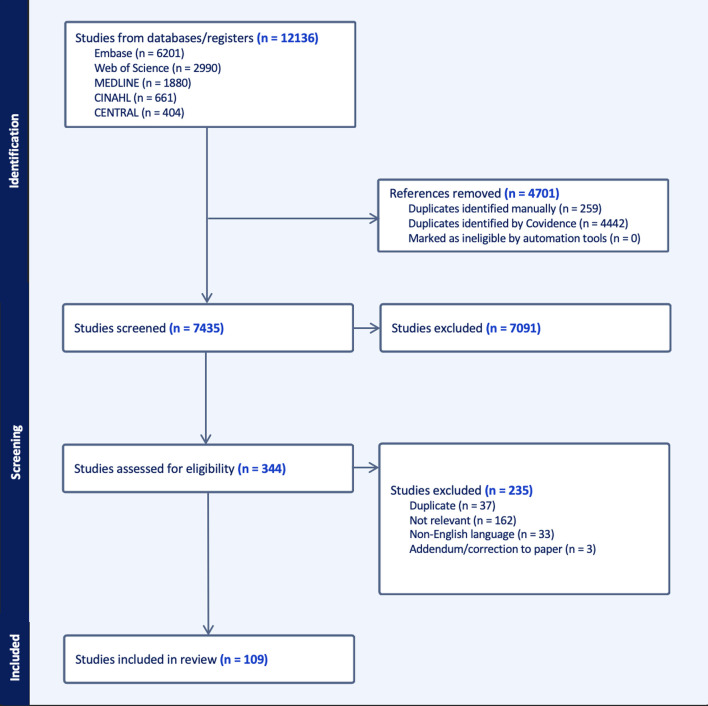
Flow diagram for process of literature search, screening, inclusion, and exclusion of articles.

The initial search yielded 12,136 articles. There were 4,442 duplicates automatically identified and removed, resulting in 7,694 items for screening. AMM and AM each independently screened the titles and abstracts using inclusion and exclusion criteria, with KP being a third independent reviewer to resolve conflicts. There were an additional 259 articles manually identified as duplicates. There were 344 articles that met criteria for full-text screening, which was subsequently performed by KP. Common reasons for exclusion included: not having a definition of polypharmacy, further duplicates identified, or abstracts of articles otherwise already included. There were 109 articles that were eligible for inclusion in our review and data were thereafter extracted. The outline of the search and screening process is outlined in Figure 1.

Details including author, year of publication, country, article type, type of arrhythmia (AF or AFL or both), clinical context, and the definition of polypharmacy used were recorded and are presented in Table 1 [27, 42, 65–155].

**Table 1:** Articles Identified That Included the Definition of Polypharmacy in AF/AFL

Author	Year	Country	Article type	Arrhythmia	Clinical context	Definition of polypharmacy used in AF/AFL
Amrouch et al [22]	2023	Multiple	Review	AF	Multiple	≥ 2 drugs
Chen et al [27]	2020	USA	Retrospective	AF	Multiple	Polypharmacy: ≥ 5 concurrently active medication prescription; substantial polypharmacy: ≥ 10 active prescriptions
Corica et al [65]	2025	Multiple	Analysis	AF	Multiple	≥ 5 drugs
Guo et al [18]	2022	China	Cross-sectional	AF	Multiple	≥ 5 long-term prescription medications
Harada [66]	2023	Japan	Analysis	AF	Multiple	≥ 5 medications
Koziel et al [5]	2020	Balkan countries	Analysis	AF	Multiple	≥ 5 prescription drugs
Grymonprez et al [50]	2024	Belgium	Prospective	AF	Multiple	≥ 5 prescription medications
Liu et al [67]	2025	Multiple	Analysis	AF	Outpatient	≥ 5 prescription medications
Ng et al [21]	2025	Multiple	Analysis	AF	Inpatient	No explicit definitions, assessed as one of the eight individual domains of the MPI
Amrouch et al [68]	2025	Multiple	Review	AF	Multiple	Not defined autonomously
Alberts et al [69]	2025	USA	Retrospective	AF	Multiple	1–4 medications, 5–9 medications, and ≥ 10 medications
Zheng et al [70]	2025	Multiple	Analysis	AF	Multiple	Not defined autonomously
Weng et al [71]	2024	USA	Retrospective	AF	Outpatient	> 5 medications
Liu et al [72]	2024	China	Prospective	AF	Inpatient	≥ 7 prescribed drugs
Catalaniet al [73]	2024	Italy	Prospective	AF	Multiple	≥ 5 medications
Goodman et al [74]	2024	Multiple	Review	AF	Multiple	≥ 5 medications
Yamamoto et al [75]	2024	Japan	Analysis	AF	Multiple	≥ 5 drugs
De Vincentis et al [76]	2024	Italy	Analysis	AF	Inpatient	≥ 5 medications
Salmasi et al [77]	2024	Canada	Retrospective	AF	Multiple	≥ 5 medications not including OAC
Corica et al [78]	2024	Italy	Review	AF	Multiple	≥ 5 drugs
Bischofet al [79]	2024	Austria	Retrospective	AF	ED	5 or more systemically active drugs
Amrouchet al [22]	2024	Sweden	Retrospective	AF	Multiple	Polypharmacy: 5–9 medications; excessive polypharmacy: ≥ 10 medications
Kim et al [28]	2024	Korea	Retrospective	AF	Multiple	≥ 5 drugs
Shimazaki et al [14]	2024	Japan	Retrospective	AF	Inpatient	≥ 6 medications
Shantsila et al [80]	2024	Multiple	Review	AF	Multiple	≥ 5 drugs
Slater et al [81]	2024	United Kingdom	Prospective	AF	Outpatient	5–9 prescribed medications
Akao et al [82]	2023	Japan	Analysis	AF	Multiple	≥ 5 medications
Verma et al [83]	2023	Multiple	Review	AF	Multiple	≥ 5 prescribed medications
Zhang et al [84]	2023	China	Prospective	AF	Multiple	> 5 drugs
Catalani et al [85]	2023	Italy	Prospective	AF	Multiple	≥ 5 medications
Chen et al [86]	2023	USA	Cross-sectional	AF	Outpatient	6–10 medications, ≥ 10 medications
Pierre-Louis et al [87]	2023	USA	Analysis	AF	Outpatient	12–14, ≥ 15
Martinez-Montesinos et al [88]	2023	Spain	Prospective	AF	Inpatient	≥ 5 drugs
Kotalczyk et al [89]	2022	China	Analysis	AF	Outpatient	≥ 5 medications
Giugliano [90]	2022	USA	Review	AF	Multiple	Not defined autonomously
Wang et al [91]	2022	China	Analysis	AF	ED	≥ 5 medications
Romiti et al [92]	2022	Multiple	Analysis	AF	Multiple	≥ 5 drugs
Caturano et al [93]	2022	Sweden	Review	AF	Inpatient	Moderate polypharmacy: 5–9 medications; severe polypharmacy: > 9
Tsagkaris et al [19]	2022	Greece	Analysis	AF	Inpatient	> 4 daily drugs
Yamashita et al [94]	2022	Japan	Analysis	AF	Outpatient	≥ 5 medications
Honda et al [95]	2022	Japan	Retrospective	AF	Inpatient	< 5, 6–9, ≥ 10 medications
Kotalczyk et al [96]	2022	China	Analysis	AF	Outpatient	≥ 5 medications
Alberts, M. et al [97]	2022	USA	Retrospective	AF	Multiple	5–9, or ≥ 10 concurrent medications
Li et al [98]	2022	Australia	Retrospective	AF	Inpatient	≥ 5 medications
Takamoto et al [99]	2021	Japan	Retrospective	AF	Outpatient	≥ 5 medications
Yokoyama et al [17]	2021	Japan	Retrospective	AF	Outpatient	≥ 5 prescribed drugs
Laliberte et al [100]	2021	USA	Retrospective	AF	Outpatient	≥ 5 medications
Fujisawa et al [101]	2021	Japan	Retrospective	AF	Outpatient	≤ 3, 4–6, 7–9, ≥ 10
Berger et al [102]	2021	USA	Retrospective	AF	Outpatient	5–9, ≥ 10 medications
Mongkhon et al [103]	2021	United Kingdom	Prospective	AF	Outpatient	≥ 5 medications
Millenaar et al [104]	2021	Multiple	Analysis	AF	Multiple	5–8, ≥ 9
Lip et al [105]	2021	USA	Analysis	AF	Multiple	≥ 6 medications
Grandone et al [106]	2021	Italy	Review	AF	Multiple	Multiple medication use
Kubas et al [107]	2020	Malaysia	Retrospective	AF	Outpatient	≥ 5 medications
van den Dries et al [108]	2020	United Kingdom	Retrospective	AF	Outpatient	≥ 5 drugs
Grymonprez et al [109]	2020	Belgium	Review	AF	Multiple	Not defined autonomously
Momo et al [110]	2020	Japan	Retrospective	AF	Not defined	> 6 medications
Mentias et al [15]	2020	USA	Retrospective	AF	Not defined	Low polypharmacy: 3 generic medications; moderate polypharmacy: 4–8 medications; severe polypharmacy: ≥ 9 medications
Gallagher et al [29]	2020	Australia	Review	AF	Multiple	Not defined autonomously
Proietti et al [111]	2020	USA	Analysis	AF	Inpatient	≥ 5 drugs
Harskamp et al [112]	2019	USA	Analysis	AF	Multiple	≥ 5 drugs
Hohmann et al [113]	2019	German	Retrospective	AF	Multiple	≥ 7 substances
Kim et al [114]	2019	Korea	Analysis	AF	Multiple	≥ 5 drugs
Martinez et al [115]	2019	USA	Retrospective	AF	Not defined	≥ 5 prescription medications; substantial polypharmacy: ≥ 10 medications
Rodriguez-Bernal et al [116]	2018	Spain	Retrospective	AF	Multiple	≥ 6 medications
Shaikh et al [117]	2018	Australia	Review	AF	Multiple	≥ 5 drugs
Paciullo et al [118]	2018	Italy	Analysis	AF	Multiple	≥ 5 drugs
Pandya et al [119]	2018	Australia	Prospective	AF	Inpatient	> 4 medications
Sabbag et al [120]	2018	USA	Review	AF	Multiple	The concomitant use of multiple different drugs
Sommerauer et al [121]	2017	Germany	Review	AF	Multiple	≥ 5 drugs
Lobos-Bejarano et al [122]	2017	Spain	Analysis	AF	Multiple	≥ 7 pills/day other than AVK
Brais et al [123]	2017	Canada	Retrospective	AF	Inpatient	At least 5 prescribed medications
Mohammed et al [124]	2017	Qatar	Retrospective	AF	Outpatient	≥ 6 medications
Mazzone et al [125]	2016	Italy	Retrospective	AF	Inpatient	≥ 5 drugs
Parks et al [126]	2016	USA	Review	AF	Multiple	Prescription of multiple medications
Piccini et al [127]	2016	USA	Analysis	AF	Multiple	0–4, 5–9, and ≥ 10 medications
Alexander et al [42]	2019	Multiple	Analysis	AF and AFL	Multiple	≥ 5 medications
Alagiakrishnan et al [45]	2019	USA	Review	AF and AFL	Multiple	≥ 5 medications
Proietti et al [128]	2016	Multiple	Analysis	AF	Multiple	Moderate polypharmacy: 5–7 medications; severe polypharmacy: 8+
Wang et al [129]	2016	Australia	Cross-sectional	AF	Outpatient	Minor polypharmacy: 5–9 medications; major polypharmacy ≥ 10 medications
Turagam et al [130]	2015	USA	Review	AF	Multiple	Increased usage of prescription and non-prescription medications
Granziera et al [131]	2015	USA	Review	AF	Multiple	≥ 5 drugs or at least 1 drug with known interference with oral anticoagulant therapy
Sanders et al [132]	2012	USA	Retrospective	AF	ED	≥ 5 more prescription or nonprescription medications
Gasse et al [133]	2005	United Kingdom	Prospective	AF	Outpatient	More than 4 prescription drugs including warfarin
Bai et al [134]	2025	China	Cross-sectional	AF	Inpatient	Polypharmacy: 5–9 drugs; excessive polypharmacy: ≥ 10 drugs
Corica et al [135]	2024	China	Analysis	AF	Multiple	≥ 5 drugs
Nicolau et al [136]	2024	USA	Review	AF	Multiple	≥ 5 medications
Jaspers Focks et al [43]	2016	Multiple	Analysis	AF and AFL	Multiple	≥ 5 drugs
Kim et al [137]	2023	Korea	Retrospective	AF	Not defined	≥ 5 drugs
Chen [46]	2016	USA	Analysis	AF and AFL	Multiple	≥ 5 prescription medications
Ãlvarez-Pinheiro et al [138]	2022	Spain	Retrospective	AF	Not defined	Polypharmacy: ≥ 5 drugs; extreme polypharmacy: ≥ 10 drugs
Amrouch et al [139]	2022	Sweden	Analysis	AF	Multiple	Mild polypharmacy: 5–10 drugs; high polypharmacy: >10 drugs
Boriani et al [140]	2022	Europe	Analysis	AF	Multiple	≥ 5 drugs
Grymonprez et al [141]	2022	Belgium	Prospective	AF	Multiple	Polypharmacy was 5–9, hyperpolypharmacy ≥ 10 drugs
Stefil, M. et al [142]	2022	Multiple	Analysis	AF	Multiple	≥ 5 medications
Voß et al [143]	2021	Europe	Retrospective	AF	Not defined	≥ 5 medications
Button et al [144]	2021	Multiple	Analysis	AF	Multiple	≥ 3 baseline medications
Alam et al [145]	2020	USA	Retrospective	AF	Multiple	≥ 5 active prescriptions
Marinelli et al [146]	2019	Italy	Prospective	AF	Inpatient	≥ 5 drugs
Shantha et al [147]	2019	Australia	Analysis	AF	Multiple	Moderate polypharmacy: 5–9 medications; severe polypharmacy: > 9 medications
La Rovere & Traversi [148]	2018	USA	Retrospective	AF	Not defined	0–5, 6–10, ≥ 11 unique active ingredients
Nissen Bonde et al [149]	2018	Multiple	Review	AF	Multiple	≥ 5 medications
Hung et al [150]	2016	Denmark	Retrospective	AF	Multiple	≥ 5 medications
Hughes & Lip [151]	2013	Taiwan	Cross-sectional	AF	Inpatient	> 4 kinds of drugs
Bolt et al [152]	2007	Multiple	Review	AF	Multiple	Not defined autonomously
Liu et al [153]	2023	Canada	Cross-sectional	AF	Outpatient	≥ 5 medications
Sa et al [154]	2023	China	Prospective	AF	Inpatient	≥ 7 medications
Chen et al [20]	2023	Brazil	Cross-sectional	AF	Outpatient	5–9 medications
Dobrica et al [155]	2023	China	Retrospective	AF	Multiple	Not defined, used MRCI instead
Amrouch et al [22]	2020	Romania	Retrospective	AF	Inpatient	≥ 5 medications

AF: atrial fibrillation; AFL: atrial flutter; ED: emergency department; MPI: Multidimensional Prognostic Index; MRCI: Medication Regimen Complexity Index; OAC: oral anticoagulant.

## Results

Of the 109 articles identified and extracted, 32% (n = 35) were retrospective, 12% (n = 13) were prospective, 6% (n = 7) were cross-sectional, 19% (n = 21) were reviews, and 30% (n = 33) were analyses which included meta-, *post hoc*, and subgroup analyses. Most articles (96%, n = 105) discussed AF only, 4% (n = 4) discussed AF and AFL concurrently without differentiation, and no articles discussed AFL exclusively. We also examined the clinical context in which definitions were used: 3% (n = 3) examined emergency department patients, 16% (n = 18) examined inpatients, 19% (n = 21) examined outpatients, and 55% (n = 60) examined patients across multiple clinical settings. Clinical context was not defined in 6% of articles (n = 7).

**Figure 2:**
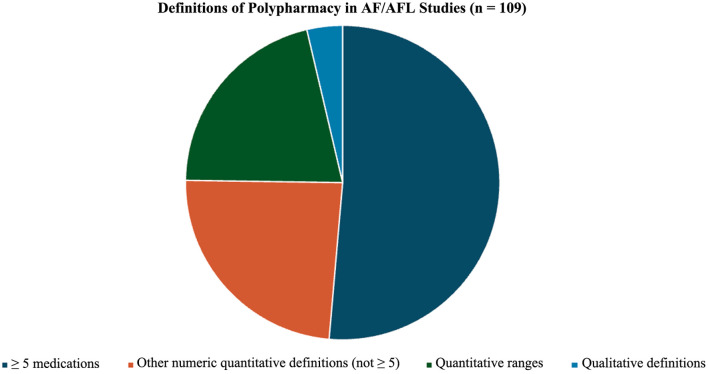
Pie chart outlining various definitions of polypharmacy extracted.

Most of the articles used a quantitative definition of polypharmacy (n = 105) with most articles using a threshold in their definition. The most common threshold was the use of ≥ 5 medications (n = 56), which was the most common definition of polypharmacy found in our review. A total of 21% of articles (n = 23) stratified polypharmacy into moderate or severe polypharmacy with differing ranges for each. A total of four articles used a qualitative definition for polypharmacy. Figure 2 summarizes the different definitions used across the extracted articles.

Articles were also assessed as to whether over-the-counter (OTC) medications or supplements were included in the definition. However, this was inconsistently reported. Minimal articles in this review attempted to assess the treatment of polypharmacy in this patient population.

## Discussion

### Common definitions of polypharmacy

Quantitative, threshold-based definitions of polypharmacy were frequent, with the most common example being the concurrent use of ≥ 5 medications. Although this definition was used by 51% (n = 56) of articles examined, there were many other numerical cutoffs used to define polypharmacy. This variety in thresholds may reflect regional and cultural differences. Shimazaki et al acknowledged that although the most common definition of polypharmacy in many different countries is the use of ≥ 5 medications, the Japanese definition of polypharmacy is the use of ≥ 6 medications [14]. A binary definition with a numeric threshold is advantageous because it provides a clear cut-off that supports consistency and comparison between different studies. Clinically, it allows providers to quickly screen and assess for the presence of polypharmacy and prompts a more thorough medication review and focus on de-prescribing initiatives.

A total of 21% of articles (n = 23) used ranges for varying levels of polypharmacy. For example, Mentias et al stratified polypharmacy, defining low polypharmacy as ≤ 3 medications, moderate polypharmacy as 4–8 medications, and high polypharmacy as ≥ 9 medications [15]. The use of ranges implies that polypharmacy exists on a continuum rather than a binary scale. The granularity offered by using ranges can better stratify patients who are at the highest risk of polypharmacy-related adverse effects. However, this approach can be nuanced and challenging to interpret at the bedside.

Terminology was inconsistent across the literature. Two over-arching terms used were “medications” and “drugs.” Though these terms are used interchangeably, “medications” typically refer to disease-specific prescribed agents while “drugs” may additionally include OTC products, herbal remedies, and supplements [16]. There was also variation in how specific the definitions used were. For example, the definition of polypharmacy in the article by Yokoyama et al was ≥ 5 prescribed medications [17]. Some articles specified frequency or duration in their definition. For example, Tsagkaris et al defined polypharmacy as the use of more than four daily drugs, while Guo et al defined it as the use of ≥ 5 long-term prescription medications [18, 19].

### Moving beyond numeric definitions

Numeric definitions of polypharmacy do not account for medication appropriateness, complexity, or associated risks. Only four articles used qualitative measures, highlighting the predominant use of numeric definitions of polypharmacy. Qualitative definitions can be beneficial as they suggest that there is no clinically meaningful numerical cut-off in which polypharmacy becomes harmful; rather, the impact of multi-medication regimens is individualized and dependent on patient-specific factors.

Only two articles in our review used indices to define polypharmacy [20, 21]. Index polypharmacy incorporates the use of well-established tools, such as Beer’s criteria and STOPP/START index, to reduce adverse drug events, elevate quality of care, prevent inappropriate prescriptions, and identify potential omissions that should be considered [8, 9]. A Swedish study that assessed patients with AF and one additional comorbidity found that potentially inappropriate prescribing (PIP) was highly prevalent. This finding underscores how index-based polypharmacy definitions capture nuance beyond drug counts and more accurately reflect the clinical consequences of polypharmacy [22].

The Medication Regimen Complexity Index (MRCI) uses a score for complexity that considers dosage forms, dosing frequency, and additional administration instructions with higher scores being associated with increased hospitalizations, readmissions, and medication non-adherence [23, 24]. One article used the MRCI score to evaluate medication complexity in AF and whether the initiation of oral anticoagulation therapy (OAT) was associated with increased bleeding risk [20]. It was found that at time points longer than 90 days, higher MRCI scores were associated with an increase in bleeding. This could underscore the importance of polypharmacy definitions that integrate medication regimen complexity, not only medication quantity. Notably, this article also used HAS-BLED as a covariable in comparing quartile groups on medication complexity. It showed that the highest quartile with respect to medication complexity had higher HAS-BLED scores. However, there was no comparison in predicting bleeding between the two. Although the article mentions that HAS-BLED is a traditional risk calculator, its limitation is that it does not consider medication complexity. However, this is only one article, and additional research is necessary to draw conclusions regarding the utility of MRCI-inclusive polypharmacy definitions in AF and AFL.

Current definitions of polypharmacy do not account for frequency of medications. This is relevant in the context of AF/AFL as some commonly used medications including carvedilol used for rate control, and apixaban, used for stroke prophylaxis, are twice daily medications. This undoubtedly contributes to increased medication regimen complexity but does not seem to be incorporated into definitions of polypharmacy. This supports the use of scoring systems that assess complexity of regimens such as the MRCI score.

### OTC medication, supplement use, and prescription considerations

Most articles did not mention the inclusion of OTC products and supplements in their definition of polypharmacy. A few articles recognized their failure to include such products as a limitation of their studies, and only one article included these agents in their definition of polypharmacy. As such, it is unclear whether OTC drugs or supplements should be included in the definition. However, it is well-established that these medications and supplements are often underreported and do contribute to polypharmacy [25, 26]. Clinicians should discuss OTC medications, herbal agents, and supplementation when performing medication reconciliation.

### Importance and impact of polypharmacy in AF and AFL

Polypharmacy in AF and AFL has clinical consequences that affect quality of life, morbidity, mortality, and therapy effectiveness. One article identified increased bleeding risk and HF as adverse outcomes associated with polypharmacy in elderly patients with AF [27]. Other studies found that polypharmacy in AF and AFL was associated with increase in all-cause mortality and decrease in effectiveness of rate and rhythm control, medication adherence, quality of life (assessed by the EuroQol-5 Dimension), and physical function [27–29]. Though likely confounded by multimorbidity, polypharmacy can potentiate these adverse consequences by reducing medication adherence, increasing the likelihood of drug-drug or drug-disease interactions, and adverse drug reactions [29]. This emphasizes the importance of clinicians performing regular medication reviews, optimizing medication regimens, considering active de-prescription efforts, and devising alternative management. Integrating patient-centered decision-making in these contexts is equally important. Such efforts can help balance symptom relief, pill burden, quality of life, and adverse effects in a way that is specific to individual risk profiles and preferences.

### Non-pharmacologic interventions

Lifestyle modification and treatment of underlying comorbidities is crucial in this patient population. There is growing evidence that aggressive lifestyle interventions can decrease AF recurrence. This includes weight loss, regular moderate aerobic exercise, alcohol reduction/cessation, continuous positive airway pressure (CPAP) use in obstructive sleep apnea (OSA), and aggressive treatment of HTN and DM [30]. Previously, it was believed that caffeine intake may be proarrhythmic and thus limiting caffeine intake was traditionally recommended. The DECAF trial contradicted conventional recommendations and found that in patients with AF or AFL who had a successful cardioversion, consumption of one cup of coffee a day was associated with less arrhythmia recurrence [31]. Lifestyle interventions can decrease symptom burden and recurrence, which may allow for deprescription.

Procedural interventions in AF and AFL mainly include cardioversion and ablation [32]. Electrical cardioversion has a high success rate exceeding 70%; however, maintenance of sinus rhythm depends on patient-specific factors [33]. Catheter ablation has excellent success rates and is considered first-line therapy for typical AFL and depending on the clinical scenario, may be first-line therapy in AF [34, 35]. In a randomized trial that assessed patients with at least two episodes of symptomatic AFL over 4 months, catheter ablation resulted in 80% of the cohort remaining in sinus rhythm compared to 36% of patients in the drug-treatment group after a mean follow-up of 21 months [36]. Due to recent evidence, ablative therapy is particularly significant in the context of polypharmacy. The ALONE-AF trial demonstrated that among patients that received ablative therapy for AF/AFL without evidence of recurrence at 1 year, discontinuation of OAT was associated with a lower incidence of the composite endpoint of stroke, systemic embolism, and major bleeding [37]. OAT contributes substantially to polypharmacy burden in AF/AFL, and this trial paves the way for avenues to deprescribe.

LAAO is used to prevent embolic events in patients with contraindications to long-term AC [38]. The PROTECT AF, PREVAIL, and PRAGUE-17 trials demonstrated that LAAO was non-inferior to long-term AC [38–40]. From a polypharmacy perspective, patients may require multiple other medications post procedure. Antibiotics are prescribed for bacterial endocarditis prophylaxis [41]. Warfarin and aspirin are used for 45 days, followed by once daily clopidogrel and aspirin for 6 months and lifelong aspirin therapy thereafter [41]. Therefore, this medication regimen must be taken into consideration when assessing polypharmacy and associated risks in patients who are being considered for LAAO.

Though lifestyle modifications, procedural interventions, and LAAO are not pharmacologic treatments, exploration of these tools can be used on a case-by-case basis to decrease medication burden for patients, particularly those at highest risk of adverse events from polypharmacy.

### Contrasting polypharmacy in AF and AFL

Our review intended to analyze the definition of polypharmacy in both AF and AFL as reflected by our search process. The rationale behind this was that both arrhythmias have very similar etiology, risk factors, and pharmacologic management (rate control, rhythm control, and AC). However, there were only four articles that included patients with AFL. Two of these articles were analyses of the ARISTOTLE trial which included patients with AF and AFL without stratification [42–44]. Another meta-analysis did not specifically look at AFL but included a study in which patients with both AF and AFL were recruited [45]. The fourth article used another database to graphically depict a prevalence of AF and AFL [46]. All four of these articles did not isolate AF and AFL diagnoses and there were no articles that looked at AFL independently of AF. Our results primarily apply to defining polypharmacy in AF. There are limited AFL-specific data which makes it difficult to draw conclusions regarding AFL independently.

Although AF and AFL are similar diagnoses with overlapping risk factors, comorbidities, and pharmacologic treatment options, it is important to distinguish the definition of polypharmacy as there are notable differences in management and response to therapy. As previously discussed, catheter ablation in most patients with typical AFL is extremely successful and can be considered curative [34, 35]. In contrast, catheter ablation for AF was historically reserved for symptomatic cases refractory to pharmacotherapy, although newer guidelines support ablation as a first-line therapy in patients with symptomatic paroxysmal AF who failed or do not want pharmacotherapy, younger patients with no or few comorbidities, and heart failure with reduced ejection fraction (HFrEF) patients. Despite this, catheter ablation for AF remains associated with lower long-term success when compared to AFL [47, 48]. This suggests that AFL patients may be less reliant on long-term pharmacotherapy compared to AF patients, resulting in differences in polypharmacy prevalence between the two populations.

### AC considerations in the context of polypharmacy

AC therapy is crucial in the treatment of AF and AFL to prevent thromboembolic disease. Numerous studies in our review discussed polypharmacy with particular concern for AC due to drug-drug interactions and increased adverse drug events. With the introduction of direct oral anticoagulants (DOAC), guidelines have rapidly changed and DOACs are preferred over warfarin for most patients. Warfarin use carries significant side effects, including a high likelihood of bleeding and multiple drug and food interactions secondary to its cytochrome P450 enzyme-dependent hepatic metabolism. Additionally, warfarin requires close titration of international normalized ratio (INR) [49]. An analysis of the ORBIT-AF trial found that the time in goal therapeutic range of INR (2–3) was suboptimal in patients with AF in the US [49]. Furthermore, those at highest risk of stroke and bleeding were least likely to be in the therapeutic range.

Multiple studies compared DOACs with warfarin in patients with polypharmacy and AF. Grymonprez et al compared AF patients who had polypharmacy, defined as ≥ 5 drugs, to those without polypharmacy [50]. Among AF patients with polypharmacy, they compared those initiating non-vitamin K antagonist oral anticoagulants (NOACs) and vitamin-K antagonists (VKAs). Patients with polypharmacy had an increased risk of stroke, major bleeding, and all-cause death compared to patients without polypharmacy. When NOAC and VKA use was compared within the polypharmacy group, NOACs had a lower risk of stroke, major bleeding, and all-cause mortality. These findings demonstrate that when starting AC for AF or AFL, providers must not only consider the presence of polypharmacy in their patients, but also the type of AC being initiated. Most patients should be placed on DOACs, unless there is a clear indication for warfarin.

As previously mentioned, the ALONE-AF trial showed that in patients without atrial arrhythmia recurrence 1 year post ablation, it may be reasonable to discontinue AC [37]. Additionally, LAAO can also be considered to avoid AC therapy but does have a caveat—patients are required to have a 45-day course of AC or dual antiplatelet therapy (DAPT) followed by indefinite single antiplatelet therapy. Although this does not reduce the number of medications, exchanging AC therapy with antiplatelet therapy can mitigate the bleeding risk.

### Strategic medication use for comorbidities

The most common comorbidities in patients with AF and AFL include CHF, CAD, HTN, myocardial infarction (MI), chronic kidney disease (CKD), OSA, and hyperthyroidism [51]. These conditions typically require pharmacotherapy, increasing polypharmacy risk. HFrEF is of particular concern as guideline-directed medical therapy (GDMT) entails the use and optimization of four medication classes including beta-blockers, renin-angiotensin-aldosterone system inhibitors, mineralocorticoid receptor antagonists (MRAs), and sodium-glucose cotransporter 2 inhibitors (SGLT2is) [52]. Additionally, newer research is investigating the utility and benefit of glucagon-like peptide-1 receptor agonists. Consequently, patients with HFrEF and AF/AFL are exposed to polypharmacy, even before accounting for their arrhythmia-directed therapy [53]. Similarly, the management of CAD, diabetes, and HTN necessitates the use of statins, antiplatelet, glucose-lowering, and anti-hypertensive therapies, further compounding the risk of polypharmacy.

In this context, strategic medication selection is critical to address both arrhythmia management and underlying comorbidities when able. For example, though beta-blockers are not first-line therapies for HTN, their use in AF/AFL serves the dual purpose of blood pressure control and rate control. Dual-purpose use and mindful prescribing in this manner could decrease the risk of polypharmacy.

The polypill, a multi-drug therapy packed into one pill, is an initiative to help decrease pill burden and reduce regimen complexity. Though there is little evidence of polypills in AF/AFL, there have been extensive benefits demonstrated in cardiovascular disease including decreased incidence of major adverse cardiac events (MACEs), improved medication adherence, increased rate of appropriate medical therapy use, and reduced cost, all without an increase incidence of adverse events [54–57]. A polypill strategy could be similarly applied to AF/AFL patients with evidence of polypharmacy, especially in the context of multimorbidity.

### Individualized pharmacotherapy, therapy de-escalation, and deprescription initiatives

There are many patient-specific considerations in the context of pharmacotherapy and polypharmacy with atrial arrhythmias. This pertains to their presentation, symptomology, age, weight, comorbidities, and kidney function that may preclude patients from different treatments. For example, with respect to AC therapy, apixaban is the most commonly used DOAC and there are criteria for dose-reduction as a strategy to lower bleeding risk. This includes two of: weight < 60 kg, age ≥ 80 years, or serum creatinine ≥ 1.5 mg/dL. As demonstrated by the RACE II trial, patients who are asymptomatic, a lenient heart rate goal of ≤ 110 did not differ from stricter goals [58]. Therefore, patients with heart rates at goal may forego rate control agents entirely, presenting an additional avenue for deprescription.

Another opportunity for medication de-escalation is in patients with AF and CAD. In AF patients with chronic, stable CAD, aspirin and OAC is associated with worse outcomes in comparison with OAC monotherapy [59]. In addition, in AF patients with CAD who had either acute coronary syndrome (ACS) or percutaneous coronary intervention (PCI), an antithrombotic regimen that included a P2Y_12_ inhibitor and apixaban yielded less bleeding events and less hospitalizations without an increase in ischemic events when compared to a regimen that included a VKA, aspirin, or both [60]. As such, patients with stable chronic CAD may benefit from medication de-escalation.

Medication non-adherence increases in prevalence with polypharmacy and medication complexity [61]. Simplification of medication regimens may increase compliance. For example, patients that miss their evening dose of apixaban or carvedilol do not achieve the full therapeutic benefit. This can prompt providers to seek alternative agents that are once-a-day medications such as rivaroxaban or metoprolol succinate to ease medication burden, increase compliance, and allow patients to attain full therapeutic coverage.

When performing medication reconciliation, providers should critically reassess the ongoing indication, duration, and derived benefit of each and every medication. Many medications are initiated for acute or time-limited indications but are never formally discontinued once those problems have improved or resolved. This exposes patients to unnecessary therapies and polypharmacy. Common examples include antihistamines for transient allergic symptoms, proton-pump inhibitors, electrolyte supplements, antidepressants, multivitamins, temporary bowel regimens, herbal supplements, and probiotics [62, 63]. Because these are often considered benign or low risk, discontinuation of these medications is often dismissed. However, re-evaluation and discontinuation of these medications serve as practical, low-risk opportunities for improving medication burden and reducing polypharmacy.

### Limitations of current definitions of polypharmacy

There are many limitations that arise when trying to define polypharmacy in AF and AFL. Firstly, there are various different definitions. Though some definitions are more common than others, the lack of standardization prevents accurate comparison and assessment of polypharmacy prevalence rates in AF and AFL. Furthermore, only one extracted article included OTC medications and supplements in its definition of polypharmacy in AF and AFL. However, OTC medications and supplements can meaningfully contribute to drug-drug interactions, increased bleeding risk, and increased medication complexity [64]. Most definitions also fail to account for medication regimen complexity including dosing frequencies, treatment durations, or changes to medication burden over time. Only one of our extracted articles integrated such concepts in its assessment of polypharmacy, by using the MRCI. Additionally, existing definitions of polypharmacy in AF and AFL do not provide clinical context to allow distinction between appropriate and inappropriate polypharmacy. Finally, we acknowledge that the literature includes evidence that pertains predominantly to AF and that studies that discussed AFL in isolation were limited.

### Future directions

To evaluate the true implications of polypharmacy in AF and AFL, a standardized definition is needed. We recognize that quantitative definitions have numerous limitations; however, they are simple, clinically effective, and the vast majority of articles used a quantitative threshold for polypharmacy in this patient population. A threshold of ≥ 5 medications is not likely helpful to prescribing providers as most patients with AF and AFL are taking ≥ 5 medications at the time of diagnosis. There is ongoing debate regarding the definition of polypharmacy in this population; however, if a numeric cutoff is utilized, we suggest increasing the cutoff to 10 or more medications. Calls for disease-specific polypharmacy definitions already exist in the literature. One example is HF, for which some suggest changing the definition of polypharmacy to ≥ 10 medications, due to the heavy medication burden of GDMT [53].

There are many directions for future research. As previously discussed, none of the studies examined polypharmacy in AFL alone, and AFL needs to be examined independently. Additionally, future studies should incorporate indices such as the MRCI, Beers criteria, and STOPP/START criteria in AF and AFL polypharmacy research. An additional avenue of future research is direct comparison of bleeding risk and adverse events using risk calculators such as the HAS-BLED with indices like the MRCI. Finally, patient-centered outcomes were scarce throughout our review. Pharmacotherapy must be individualized, and prescribing patterns should incorporate pill burden, adherence, symptom control, quality of life, and goals of care.

Minimal articles in this review attempted to assess the treatment of polypharmacy in this patient population. Therefore, future investigation should examine trials with structured, active deprescribing efforts, utilizing a multidisciplinary approach. Additional evaluation of polypill strategies, dual-purpose use, and mindful prescribing for better management of comorbidities in AF/AFL patients is also crucial.

## Conclusion

Polypharmacy is prevalent in patients with AF and AFL and can lead to falls, bleeding, drug-drug interactions, adverse drug-related events, medication non-adherence, and decreased treatment efficacy. To mitigate polypharmacy and its adverse effects, patients with AF and AFL should be counseled on aggressive lifestyle modification, and providers should strategically utilize medications. Based on our review, the most common definition of polypharmacy in this patient population was the use of ≥ 5 medications. However, this definition is likely not helpful to prescribing providers as most of these patients are already on > 5 medications. There is ongoing debate regarding the definition of polypharmacy in this population. If a numeric cutoff is utilized, we propose increasing it to 10 or more medications, which has also been suggested in the HF population. More importantly, regardless of how we define polypharmacy in AF or AFL, it is crucial that providers continually assess each and every medication to ensure that the benefits outweigh the risks.

## Supplementary Material

Suppl 1 Search documentation.

## Data Availability

The authors declare that data supporting the findings of this study are available within the article.

## Abbreviations

AC: anticoagulation
ACS: acute coronary syndrome
AF: atrial fibrillation
AFL: atrial flutter
CAD: coronary artery disease
CKD: chronic kidney disease
CPAP: continuous positive airway pressure
DAPT: dual antiplatelet therapy
DOACs: direct oral anticoagulants
GDMT: guideline-directed medical therapy
HF: heart failure
HFrEF: heart failure with reduced ejection fraction
HTN: hypertension
INR: international normalized ratio
LAAO: left atrial appendage occlusion
MACEs: major adverse cardiac events
MI: myocardial infarction
MRAs: mineralocorticoid receptor antagonists
MRCI: Medication Regimen Complexity Index
NOACs: non-vitamin K antagonist oral anticoagulants
OAT: oral anticoagulation therapy
OSA: obstructive sleep apnea
OTC: over-the-counter
PCC: population–concept–context
PCI: percutaneous coronary intervention
PIP: potentially inappropriate prescribing
SGLT2is: sodium-glucose cotransporter 2 inhibitors
START: Screening Tool to Alert to Right Treatment
STOPP: Screening Tool of Older Person’s Prescriptions
VKAs: vitamin-K antagonists
